# Identification of plasma inflammatory biomarkers for Alzheimer’s disease reveals IFN-γ as a regulator of ACSL1-mediated microglia phenotype

**DOI:** 10.3389/fimmu.2026.1770509

**Published:** 2026-02-17

**Authors:** Ronghua Huang, Bing-Biao Lin, Zhijie Lu, Yixuan Hao, Chenrui Li, Zixian Lin, Yingjie Zhang, Naili Wei, Jian Chen

**Affiliations:** 1 Department of Neurosurgery, The First Affiliated Hospital of Shantou University Medical College, Shantou, Guangdong, China; 2 Laboratory of Molecular Cardiology, The First Affiliated Hospital of Shantou University Medical College, Shantou, Guangdong, China; 3 Department of Anesthesiology, The First Affiliated Hospital of Shantou University Medical College, Shantou, Guangdong, China; 4 Department of Radiation Oncology, Cancer Hospital of Shantou University Medical College, Shantou, Guangdong, China; 5 Guangdong Provincial Key Laboratory on Brain Function Repair and Regeneration, The Neurosurgery Institute of Guangdong Province, Zhujiang Hospital, Southern Medical University, Guangzhou, China; 6 Department of Obstetrics, Rongcheng Women Infant Health Care Hospital, Jieyang, Guangdong, China; 7 Department of Rehabilitation, The First Affiliated Hospital of Shantou University Medical College, Shantou, Guangdong, China

**Keywords:** Alzheimer’s disease, APOE, biomarker, IFN-gamma, inflammation

## Abstract

**Background:**

The identification of plasma biomarkers for the diagnosis of Alzheimer’s disease (AD) has been a longstanding research priority; however, few plasma biomarkers have yet been implemented in routine clinical practice.

**Methods:**

This study enrolled 141 participants, including 71 patients with AD, 44 individuals with mild cognitive impairment, and 28 cognitively healthy controls (HC). A total of 16 plasma inflammatory proteins were quantified using multiplex liquid-chip assays, and APOE genotyping was performed. The diagnostic utility of plasma proteins was assessed using the least absolute shrinkage and selection operator (LASSO) with nested cross-validation.

**Results:**

Patients with AD exhibited marked alterations in plasma inflammatory profiles, with elevated levels of IFN-γ, IL-33, and IL-18, and reduced levels of IL-7 and CCL11. Integrating inflammatory markers with clinical variables and APOE genotype substantially improved discrimination between AD and HC, increasing the area under the ROC curve from 0.863 to 0.953. Among all biomarkers, IFN-γ emerged as the most informative predictor and was significantly elevated in AD patients carrying the APOE ϵ4 allele. Analyses of single-nucleus RNA sequencing data further revealed pronounced enrichment of IFN-γ signaling in APOE4/4 AD-associated lipid droplet-accumulating microglia (LDAM), defined by high ACSL1 expression. Notably, IFN-γ stimulation enhanced ACSL1 expression in ApoE4-overexpressing HMC3 microglial cells.

**Conclusion:**

These findings provide a new perspective on the involvement of plasma inflammatory markers for AD diagnosis, and suggest a novel link between IFN-γ and APOE ϵ4-associated AD risk through modulating the ACSL1-driven pathogenic LDAM phenotype.

## 1 Introduction

Alzheimer’s Disease (AD) is a chronic, progressive neurodegenerative disorder characterized primarily by cognitive decline and neurobehavioral symptoms, accounting for 60%-80% of all dementia cases (1, 2). With China’s rapidly aging population, the crude incidence rate of AD and other dementias increased markedly from 59.8 to 204.8 per 100,000 between 1990 and 2021, marking a 242.5% increase (3). Annual expenditures associated with AD exceed $150 billion, and AD, along with other dementias, has become the fifth leading cause of death in China, posing an enormous healthcare and socioeconomic burden (3).

Early diagnosis is critical for AD patients to receive timely interventions (4). Clinically, AD is a diagnosis of exclusion, made after ruling out many non-neurodegenerative and cerebrovascular conditions (5). Current diagnostic approaches primarily rely on cognitive assessments and neuroimaging modalities (5). However, the limited availability of magnetic resonance imaging (MRI) and positron emission tomography (PET) in under-resourced regions, coupled with the inherent subjectivity of cognitive tests, contributes substantially to AD underdiagnosis (6). Recent advances suggest that cerebrospinal fluid (CSF) and blood biomarkers, particularly amyloid-β and phosphorylated Tau, may assist in identifying individuals with AD pathology (7, 8). Nonetheless, most assays remain restricted to research use and are hindered by high cost, technical complexity, or invasiveness, limiting their clinical applicability (9). Therefore, searching for biomarkers that can be easily implemented for AD diagnosis is urgently needed.

It’s well established that AD pathogenesis extends beyond neuronal dysfunction and critically involves neuroinflammatory mechanisms (10, 11). Microglia, the resident immune cells of the central nervous system, are key drivers of AD-associated neuroinflammation. Experimental studies have shown that microglia recognize and bind to Aβ plaques and extracellular neurofibrillary tangles, triggering innate immune responses (11–13). Although early inflammatory activation may facilitate the clearance of toxic aggregates, chronic microglial activation results in the production of neurotoxic mediators such as nitric oxide and reactive oxygen species, and increased secretion of pro-inflammatory cytokines (10, 11, 13). Accordingly, differences in plasma inflammatory biomarkers have been reported between AD patients and healthy controls (14–16); however, the results remain inconsistent across studies, and the diagnostic value of circulating inflammatory biomarkers requires further investigation.

The APOE ϵ4 allele is the strongest known genetic risk factor for late-onset AD. Individuals carrying one APOE ϵ4 allele have a 3- to 4-fold increased risk of developing AD, whereas homozygous ϵ4 carriers face a 9- to 15-fold higher risk (17–19). Accumulating evidence indicates that apolipoprotein E4 (ApoE4) profoundly influences microglia-mediated neuroinflammatory responses, thereby accelerating AD pathogenesis (20–22). In particular, ApoE4 has been linked to the emergence of ACSL1-driven lipid droplet-accumulating microglia (LDAM), a pathogenic microglial state capable of inducing neuronal death and promoting hyperphosphorylated Tau (p-Tau) accumulation (20). Despite these insights, the molecular pathways that contribute to ApoE4-associated inflammatory cascades and microglia phenotype reprogramming remain largely unknown.

In this study, we aimed to evaluate the diagnostic value of plasma inflammatory biomarkers for AD and to examine the relationships between key biomarkers, cognitive performance, and APOE genotypes. A predictive model for AD was constructed by integrating plasma biomarkers, clinical factors, and APOE genotype. Our findings identified IFN-γ as the most informative biomarker for AD prediction, with levels significantly elevated in APOE ϵ4 carriers. Furthermore, experiments revealed that IFN-γ modulated the LDAM phenotype by upregulating ACSL1 expression in ApoE4-overexpressing microglia.

## 2 Methods

### 2.1 Participants

This study enrolled 141 participants. All procedures were conducted in accordance with the Declaration of Helsinki and were approved by the Clinical Ethics Committee of The First Affiliated Hospital of Shantou University Medical College (2020-115-XZ2, B-2022-232). Written informed consent was obtained from all participants before inclusion. AD was diagnosed using a combination of clinical assessments, neuropsychological evaluations, and neuroimaging examinations, following the revised NINCDS-ADRDA criteria endorsed by the International Working Group in 2007. Participants were excluded if they had experienced a clinical stroke within three months before enrollment; presented with MRI-confirmed regional cerebral infarction; had autoimmune or systemic inflammatory diseases; had a history of malignancy; or had received treatment with non-steroidal anti-inflammatory drugs or corticosteroids, as such medications may influence circulating cytokine concentrations.

### 2.2 Cognitive test

Cognitive function was assessed in all participants using locally adapted versions of the Mini-Mental State Examination (MMSE, 30-point scale) and the Montreal Cognitive Assessment (MoCA). The MMSE comprised assessments of orientation, registration, recall, attention and calculation, and language. The MoCA consisted of seven domains: visuospatial/executive function, naming, memory and delayed recall, attention, language, abstraction, and orientation. All participants were allowed to use personal visual aids (e.g., corrective glasses) as needed and completed the test in their preferred language to ensure optimal performance and communication. The MMSE was administered first, followed by the MoCA to minimize potential learning or fatigue effects.

### 2.3 MRI and visual rating scales

MRI examinations were performed on a 3.0-T scanner. Comprehensive imaging sequences were used, including T1 fluid-attenuated inversion recovery (FLAIR), T2 FLAIR, T2-weighted imaging, sagittal T2 fast spin-echo, and diffusion-weighted imaging, to exclude unrelated intracranial abnormalities. MTA was independently assessed by a neurologist and a radiologist. Any discrepancies in scoring were resolved by consensus. Abnormal MTA was defined according to established age-adjusted criteria: MTA score ≥ 1 for individuals < 65 years, ≥ 2 for those 65–74 years, and ≥ 3 for individuals ≥ 75 years.

### 2.4 Blood sample collection

Peripheral blood samples were collected from all participants and processed immediately. Samples were centrifuged at 300 × g for 5 minutes at 4 °C. A 1-mL aliquot of whole blood was reserved for APOE genotyping, while the remaining 3 mL was used for plasma and peripheral blood mononuclear cell isolation. The supernatant obtained after centrifugation, corresponding to the plasma fraction, was carefully harvested and stored for subsequent quantification of inflammatory proteins.

### 2.5 Inflammatory biomarker measurements using Luminex liquid chip technology

Plasma concentrations of inflammatory biomarkers were quantified using Luminex liquid bead–based multiplex technology according to the manufacturer’s protocols. The panel included TNF-α, IFN-γ, IL-1β, IL-5, IL-6, IL-7, IL-8, IL-10, IL-13, IL-17, IL-18, IL-33, MCP-1, soluble TREM-1, TSLP, and CCL11.

### 2.6 APOE genotyping

APOE genotyping was performed using the SNaPshot Multiplex System, following procedures described previously. The assay targeted two single-nucleotide polymorphisms, rs429358 and rs7412, located in exon 4 of the APOE gene. APOE genotype was determined based on the combination of ϵ2, ϵ3, and ϵ4 alleles. Individuals carrying at least one ϵ4 allele (ϵ4/ϵ4, ϵ3/ϵ4, or ϵ2/ϵ4) were classified as APOE ϵ4 carriers, whereas all other genotypes were categorized as non-APOE ϵ4 carriers.

### 2.7 Model construction and evaluation

Classification models were constructed using least absolute shrinkage and selection operator (LASSO) regression within a nested cross-validation (CV) framework, which inherently reduces the risk of multicollinearity through coefficient shrinkage and variable selection. The inner 5-fold CV was used to determine the optimal regularization parameter (λ), and the outer 5-fold CV was applied for independent model evaluation. The dataset was randomly divided into five approximately equal folds; in each iteration, one fold was used as the test set and the remaining four folds as the training set. Predictors with missing values were excluded. Categorical variables were one-hot encoded, and all predictors were centered and scaled prior to model fitting. Model performance was assessed using receiver operating characteristic (ROC) analysis. Final models were refitted using the optimized λ identified by cv.glmnet on the full dataset.

### 2.8 Single-nucleus RNA-sequencing analysis

Single-nucleus RNA-sequencing (snRNA-seq) data from fresh-frozen frontal cortex samples of individuals with AD carrying the APOE4/4 genotype, individuals with AD carrying the APOE3/3 genotype, and age- and sex-matched cognitively normal APOE3/3 controls were retrieved from the GSE254205 dataset. Gene expression matrices were log-normalized and scaled prior to downstream analyses. Highly variable features were identified using Seurat’s variance-stabilizing transformation. Principal component analysis (PCA) was conducted on the top 2,000 highly variable genes using the RunPCA function. The top 50 principal components were then used to construct a shared nearest-neighbor graph with FindNeighbors. Dimensionality reduction was further visualized in two dimensions using the Uniform Manifold Approximation and Projection algorithm (RunUMAP). Major cell-type annotations were obtained from the original study (20).

Subsequent analyses focused specifically on microglia. To minimize inter-sample variability, Harmony was applied to correct batch effects within the microglial subset. Microglial subclusters were identified using a clustering resolution of 0.4 and were annotated according to canonical marker genes and cluster-specific transcriptional signatures. To quantify pathway activity, inflammatory response and IFN-γ response scores were computed for each major cell type and for each microglial subcluster using gene sets from the HALLMARK collection (h.all.v7.4.symbol, https://www.gsea-msigdb.org/gsea/msigdb/, **Supplementary Table 1**) and the AUCell algorithm.

### 2.9 Cell culture

HMC3 were purchased from the Procell Life Science & Technology Co. (Procell, Wuhan, China). Cells were authenticated by STR profiling and tested negative for mycoplasma contamination. HMC3 were cultured in MEM supplemented with non-essential amino acids, 10% fetal bovine serum (Sigma-Aldrich, cat#F0193, USA), and 1% penicillin/streptomycin (100 U/mL, GIBCO, cat#15140122, USA). For IFN-γ-related experiments, 3 x 10^5^ cells were seeded onto 6-well plates (Guangzhou Jet Bio-Filtration Co., Ltd., cat#TCP010006) and treated with 100 ng/mL IFN-γ (MedChemExpress, cat#HY-P7025, USA) for 24 h before RNA and protein extraction.

### 2.10 Expression of ApoE isoforms in HMC3

Lentiviral expression plasmids pLVX-Ctrl-Flag-ZsGreen-Puro, pLVX- APOE2-Flag-ZsGreen-Puro, pLVX-hAPOE3-Flag-ZsGreen-Puro, and pLVX-hAPOE4-Flag-ZsGreen-Puro were constructed by Dongze Co. Each plasmid was transfected into HEK293T packaging cells to generate high-titer lentiviral particles carrying the corresponding transgenes. Viral supernatants were collected after 48 h, filtered, and concentrated. HMC3 were then transduced with the respective lentiviruses in the presence of 8 μg/mL polybrene, followed by 2 μg/mL puromycin selection. Successful transduction was confirmed by ZsGreen fluorescence, real-time qPCR, and immunoblotting of Flag-tagged proteins.

### 2.11 RNA extraction and real-time qPCR

Total RNA was extracted by Total RNA Extraction Kit (Goonie, cat# 400-100, Guangzhou, China), and cDNA was synthesized with the HiScript III All-in-one RT SuperMix (Vazyme, cat# R333-01, Nanjing, China) using 1 μg RNA as input. Real-time qPCR was performed with the Fast Taq SYBR Green qPCR Mix (Goonie, cat# 500-102). GAPDH was used as an internal control. Primer sequences used for real-time qPCR were listed in **Supplementary Table 2**.

### 2.12 Western blot

Cells were washed with ice-cold PBS and lysed in RIPA buffer supplemented with protease and phosphatase inhibitors. Equal amounts of protein were separated by 7.5% SDS-PAGE and transferred onto polyvinylidene difluoride membranes (0.22 μm pore size; Merck Millipore, cat# ISEQ00010, Germany). Membranes were blocked with 5% skim milk for 1 h at room temperature, incubated with primary antibodies overnight at 4 °C, washed six times with TBST, and then incubated with horseradish peroxidase–conjugated secondary antibodies (1:8000) for 1 h at room temperature. Protein signals were visualized using enhanced chemiluminescence reagents on an automated imaging system (Bio-Rad ChemiDoc XRS). Primary antibodies applied in this study include antibodies against APOE4 (1:1000, CST, cat# 8941), ACSL1 (1:1000, CST, cat# 9189), Flag (1:20000, Proteintech, cat# 20543-1-AP), and GAPDH (1:10000, Proteintech, cat# 60004-1-Ig).

### 2.13 Statistical analysis

All statistical analyses and data visualizations were performed using GraphPad Prism (version 7.0) and R (version 4.4.3). Receiver operating characteristic (ROC) curve analysis and calculation of the area under the curve (AUC) for binary classification were conducted using the “pROC” package in R. Spearman’s rank correlation was applied to assess associations between continuous variables. For comparisons of continuous variables between 2 groups, either Student’s t-test or the Wilcoxon rank-sum test was used. All statistical tests were two-sided, and P < 0.05 was considered statistically significant.

## 3 Result

### 3.1 Participant characteristics

A total of 143 participants were included for analyses, with 71 patients diagnosed with AD, 44 patients with mild cognitive impairment (MCI), and 28 cognitively healthy controls (HC) in the First Affiliated Hospital of Shantou University Medical College. Participant characteristics are summarized in **Table 1**. As expected, both MMSE and MoCA scores, which reflect global cognitive performance, were markedly reduced in individuals with AD. Consistent with previous reports, patients with AD were generally older, had fewer years of formal education, and exhibited a higher proportion of APOE ϵ4 genotype. Sex distribution did not differ significantly between the AD and non-AD groups. Altogether, these demographic patterns closely mirror those described in other cohorts, supporting the representativeness and reliability of our study population.

**Table 1:** Characteristics of participants included in this study.

Characteristics	Non-AD		AD	P
	HC N=28	MCI N=44	N = 71	
Age	59.8 (10.1)	64.3 (6.47)	73.2 (7.68)	<0.001
Sex:				0.956
Female	22 (78.6%)	29 (65.9%)	49 (69.0%)	
Male	6 (21.4%)	15 (34.1%)	22 (31.0%)	
Education year	11.4 (3.96)	9.70 (3.52)	6.81 (4.68)	<0.001
APOE genotype:				<0.001
E4-	25 (89.3%)	34 (77.3%)	36 (50.7%)	
E4+	3 (10.7%)	10 (22.7%)	35 (49.3%)	
MMSE	27.6 (5.26)	26.1 (4.37)	13.1 (8.31)	<0.001
MoCA	24.1 (5.01)	21.1 (3.11)	8.97 (6.67)	<0.001
TNF-α	1.62 (0.20)	1.90 (1.01)	1.90 (1.14)	0.504
IL-6	1.89 (1.65)	1.98 (1.94)	1.18 (1.15)	0.003
IL-33	2.35 (0.06)	2.92 (1.92)	3.72 (2.25)	0.002
IL-8	5.76 (6.09)	6.25 (5.27)	4.67 (8.35)	0.247
IL-7	6.24 (2.81)	6.01 (3.62)	4.02 (3.27)	<0.001
IL-10	12.0 (3.53)	11.8 (3.45)	12.3 (4.06)	0.532
MCP-1	266 (72.9)	254 (56.5)	396 (835)	0.174
IL-1β	35.0 (8.66)	39.3 (9.27)	32.1 (6.10)	<0.001
IFN-γ	57.0 (20.2)	76.4 (16.5)	98.2 (23.0)	<0.001
IL-17	7.13 (5.04)	11.0 (6.41)	10.7 (6.25)	0.235
IL-13	452 (282)	516 (207)	589 (239)	0.015
TSLP	11.9 (3.10)	11.2 (3.71)	9.94 (3.41)	0.009
IL-5	8.82 (2.87)	10.5 (2.28)	9.82 (3.57)	0.979
TREM-1	196 (89.3)	261 (59.2)	214 (70.4)	0.091
CCL11	281 (86.5)	294 (81.8)	240 (94.6)	0.001
IL-18	145 (66.5)	157 (65.9)	265 (204)	<0.001

Continuous variables are shown as mean (standard deviation), while categorical variables are presented as counts (ratios). P values were calculated based on Student’s t-test for continuous variables and Chi-square test for categorical variables between AD and non-AD groups. The concentration unit for all plasma inflammatory proteins is pg/mL. AD, Alzheimer’s disease; MCI, mild cognitive impairment; HC, healthy controls; MMSE, Mini-Mental State Examination; MoCA, Montreal Cognitive Assessment; SD, standard deviation.

### 3.2 Correlations between inflammatory biomarkers and clinical traits

**Figure 1:**
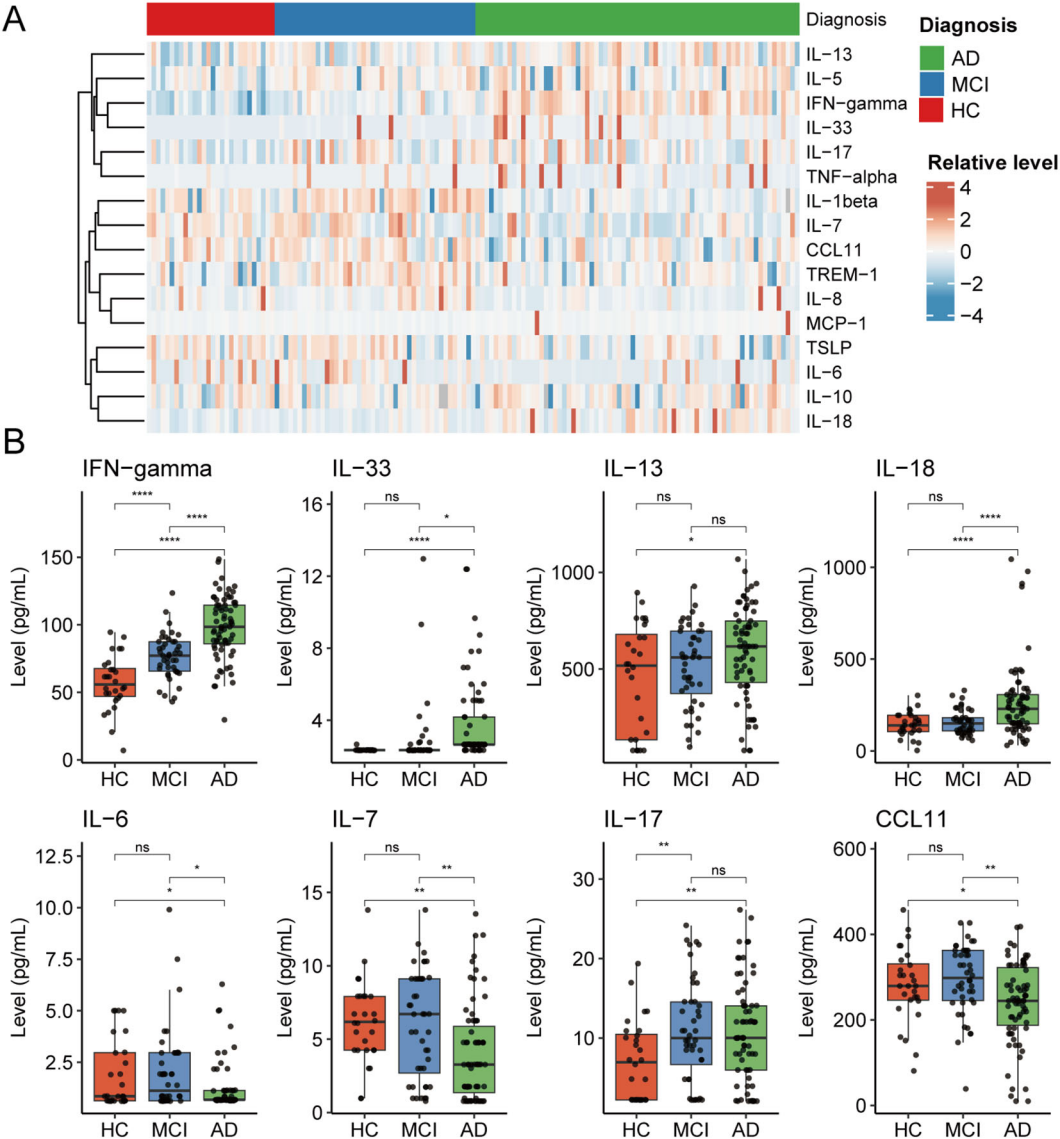
Differential expression of plasma inflammatory proteins among AD, MCI, and HC groups. **(A)** Heatmap showing the relative expression levels of 16 plasma inflammatory markers in individuals with Alzheimer’s disease (AD), mild cognitive impairment (MCI), and healthy controls (HC). **(B)** Box plots comparing the plasma levels of eight significantly differentially expressed inflammatory markers among the AD, MCI, and HC groups, including IFN-γ, IL-33, IL-13, IL-18, IL-6, IL-7, IL-17, and CCL11. ns, not significant; *P < 0.05; **P < 0.01; ****P < 0.0001.

**Figure 2:**
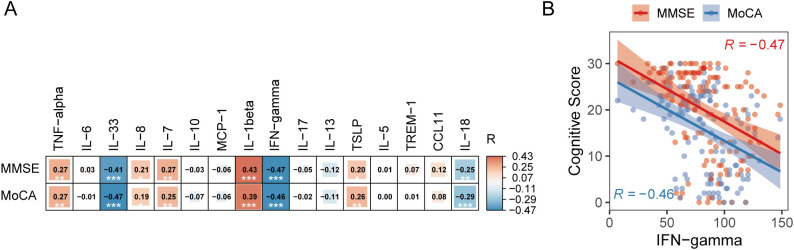
Correlations between plasma inflammatory proteins and cognitive performance. **(A)** A heatmap illustrating the Spearman correlation coefficients between 16 plasma inflammatory markers and cognitive scores, including the Mini-Mental State Examination (MMSE) and Montreal Cognitive Assessment (MoCA). Red indicates positive correlations (R > 0), whereas blue indicates negative correlations (R < 0). **(B)** A scatter plot showing the correlations between IFN-γ levels and MMSE and MoCA scores. All correlation coefficients and corresponding P values were calculated using Spearman’s rank correlation analysis. *P < 0.05; **P < 0.01; *** P < 0.001.

Next, we measured plasma levels of 16 inflammatory biomarkers (**Figure 1A**). The results revealed significant increases in IFN-γ, IL-33, and IL-18, accompanied by significant reductions in IL-7, IL-6, and CCL11 in patients with AD (**Table 1**; **Figure 1B**). We then further interrogated their associations with cognitive levels (**Figure 2A**) and observed remarkable negative correlations between IFN-γ and MMSE (R = -0.47, P < 0.001) or MOCA (R = -0.46, P < 0.001) scores (**Figure 2B**). In addition, both IL-33 and IL-18 were negatively correlated with MMSE (IL33: R = -0.41, P < 0.001; IL-18: R = -0.25, P < 0.01) or MOCA (IL33: R = -0.47, P < 0.001; IL-18: R = -0.29, P < 0.001). In contrast, higher IL-7, IL-8, and TSLP were significantly associated with higher MMSE or MOCA scores (**Figure 2A**). Together, these alterations indicate a profound dysregulation of systemic inflammatory mediators in AD, potentially reflecting an underlying shift in neuroinflammatory activity.

### 3.3 Construction of predictive models based on plasma proteins, clinical factors, and APOE genotype

**Figure 3:**
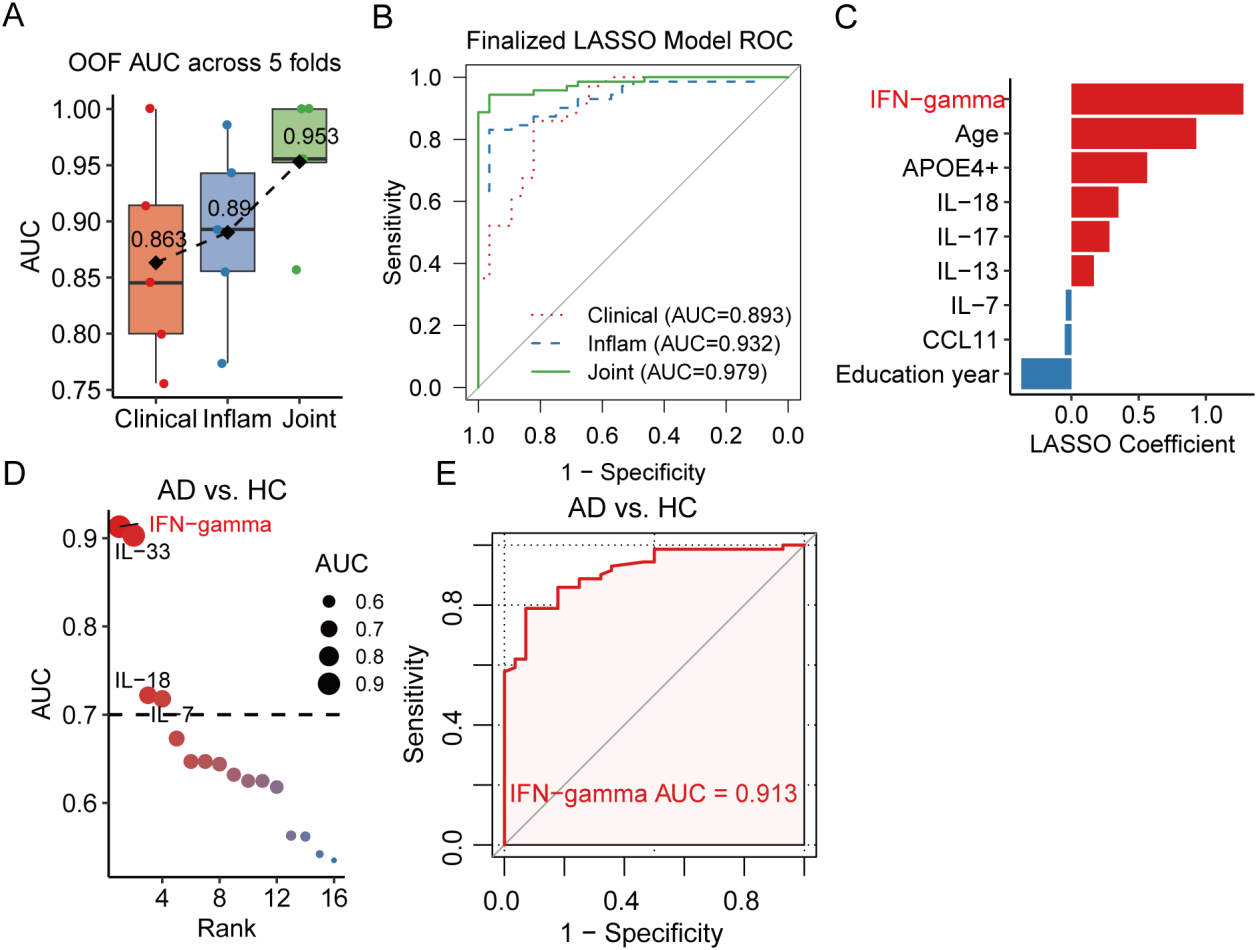
Predictive performance of clinical, inflammatory, and combined models for distinguishing AD from HC. **(A)** Box plots showing the out-of-fold area under the receiver operating characteristic curve (AUC) values from five cross-validation folds for the clinical factor model, plasma inflammatory marker model, and the combined clinical and inflammatory model in discriminating Alzheimer’s disease (AD) from healthy controls (HC). Diamonds indicate the mean AUC across the five folds. **(B)** Receiver operating characteristic (ROC) curves of the final models based on clinical factors, inflammatory proteins, and their combination, trained using the entire cohort. **(C)** Features contributing to the prediction of AD in the final combined model. Variables with regression coefficients greater than zero indicate a positive association with AD risk, whereas variables with coefficients less than zero indicate a negative association with AD risk. **(D)** Results of ROC curve analysis evaluating the diagnostic performance of the 16 inflammatory proteins in distinguishing AD from HC. **(E)** ROC curve analysis of IFN-γ for discriminating AD from HC.

We then applied the LASSO algorithm within a 5 × 5 nested CV framework to optimize λ and objectively compare model performance. As shown in **Figure 3A**, the optimized model incorporating clinical variables and APOE genotype distinguished AD from HC with a mean out-of-fold AUC of 0.863. In comparison, the model based solely on inflammatory biomarkers achieved a higher mean AUC of 0.890, and the integrated model combining clinical variables, APOE genotype, and plasma protein measurement yielded the highest mean AUC of 0.953, indicating that inflammatory biomarker alterations provide additional discriminative information beyond conventional clinical variables. Subsequently, final models were established using all included participants and demonstrated a consistent pattern of predictive performance (**Figure 3B**; **Supplementary Figure S1**).

### 3.4 Higher IFN-γ was associated with increased predictive probability of AD

Next, the inferred LASSO coefficients were used to quantify the relative contribution of each feature to the finalized joint model. As shown in **Figure 3C**, a set of protective factors, such as longer duration of education, IL-7, and CCL11, was associated with a reduced probability of AD. In contrast, several variables exerted strong risk-enhancing effects, including IFN-γ, older age, APOEϵ4 carrier status, IL-13, IL-17, and IL-18, all of which shifted model predictions toward AD when increased or present. Notably, IFN-γ exhibited the largest coefficient magnitude, indicating its dominant role in driving model predictions. In line with this, ROC analysis identified IFN-γ as the most predictive marker for AD among 16 proteins (AUC = 0.913, **Figures 3D, E**), and further demonstrated its ability to distinguish AD from MCI individuals (AUC = 0.789, **Supplementary Figure S2**). Collectively, these findings support a potential role for IFN-γ in mediating inflammatory processes in AD pathogenesis.

### 3.5 IFN-γ pathway enrichment in lipid-droplet accumulating microglia in APOE4/4 AD

**Figure 4:**
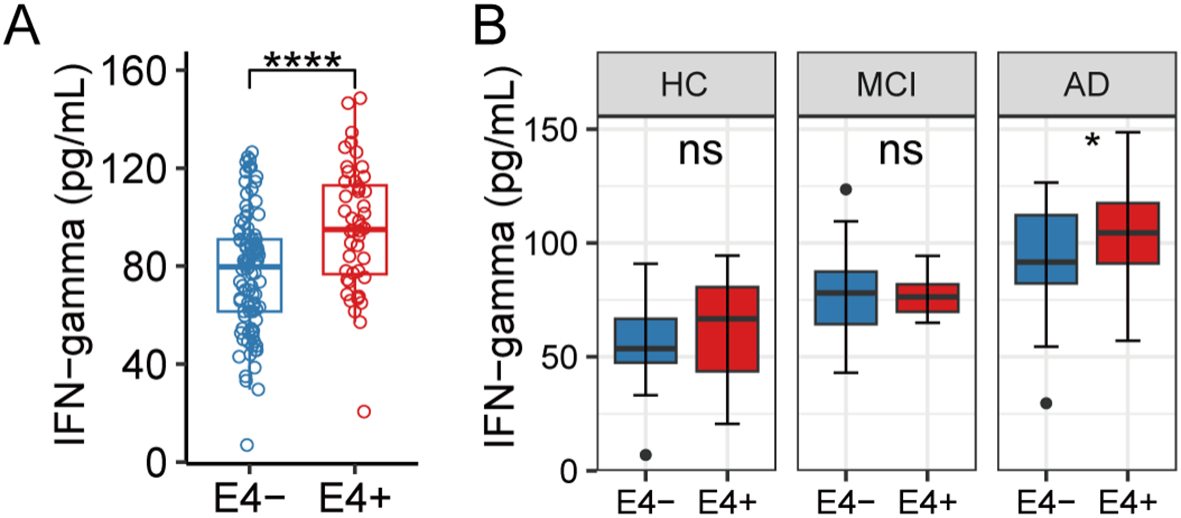
Association between APOE ϵ4 genotype and plasma IFN-γ levels. **(A)** A box plot comparing plasma IFN-γ levels between APOE ϵ4 carriers and non-carriers. **(B)** Box plots comparing plasma IFN-γ levels between APOE ϵ4 carriers and non-carriers within the healthy controls (HC), mild cognitive impairment (MCI), and Alzheimer’s disease (AD) subgroups. ns, not significant; *P < 0.05; ****P < 0.0001.

Recent studies have highlighted a strong link between neuroinflammation and APOE4 genotypes in AD. We observed significantly higher plasma IFN-γ levels in APOE ϵ4 carriers compared with noncarriers (**Figure 4A**). Subgroup analyses revealed that this elevation was specific to individuals with AD, as no significant differences were observed in HC or MCI subjects (**Figure 4B**). These findings led us to hypothesize that IFN-γ signaling may be selectively upregulated in specific brain cell types of APOE4/4 AD patients.

**Figure 5:**
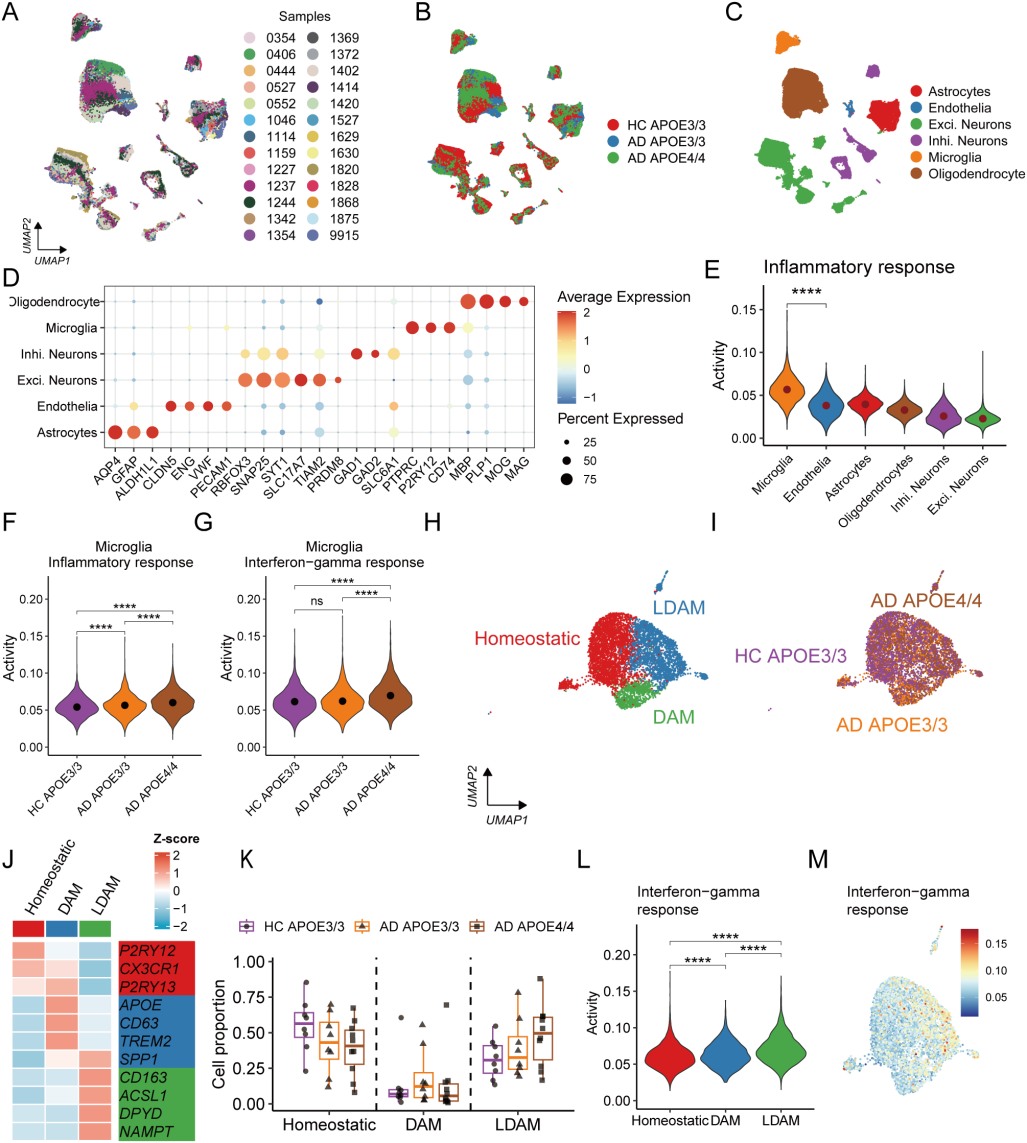
Single-cell transcriptomic landscape reveals APOE genotype- and disease-associated inflammatory alterations in microglia. Uniform Manifold Approximation and Projection (UMAP) plot showing the distribution of 100,317 cells colored by samples **(A)**, APOE genotype and disease status **(B)**, and major cell types **(C)**. **(D)** A dot plot illustrating the expression levels and proportions of marker genes for the 6 major cell types. **(E)** A box plot comparing inflammatory pathway activity across major cell types. Box plots comparing inflammatory pathway activity **(F)** and IFN-γ signaling scores **(G)** in microglia among APOE3/3 healthy controls (HC), APOE3/3 Alzheimer’s disease (AD), and APOE4/4 AD groups, respectively. **(H)** A UMAP plot showing the subcluster distribution of 6,573 microglial cells. **(I)** UMAP visualization of microglial cells colored by APOE genotype and disease status. **(J)** A heatmap showing the expression levels of marker genes across 3 microglial subclusters. **(K)** Box plots comparing the proportions of the three microglial subclusters among APOE3/3 HC, APOE3/3 AD, and APOE4/4 AD groups. **(L)** A box plot comparing IFN-γ signaling scores among the three microglial subclusters. **(M)** A featureplot showing the activity of IFN-γ response among microglial cells. Exci., excitory; Inhi., inhibitory; DAM, disease associated microglia; LDAM, lipid droplet-accumulating microglia. ns, not significant; ****P < 0.0001.

To investigate this, we analyzed published snRNA-seq data generated from 26 human cortex samples (**Figures 5A-C**), including AD patients with APOE4/4 (n = 10) or APOE3/3 (n = 8) genotypes, and cognitively normal APOE3/3 controls (n = 8) (20). Major brain cell types were annotated according to the original study and further verified using canonical marker genes (**Figure 5D**), which included astrocytes (AQP4, GFAP), endothelia (CLDN5, VWF), neurons (RBFOX3, SYT1), microglia (P2RY12, CD74), and oligodendrocytes (MBP, PLP1). Notably, microglia were the primary cell type (6.63% of all annotated cells) exhibiting significant enrichment in the inflammatory response (**Figure 5E**). Moreover, microglia from APOE4/4 AD patients displayed markedly higher activity in the inflammatory and IFN-γ pathways compared with those from APOE3/3 AD patients or HC (**Figures 5F, G**), suggesting a genotype-specific amplification of IFN-γ-driven inflammatory signaling in APOE4-associated AD.

Further subclustering of microglia identified three transcriptionally distinct subtypes: (i) disease-associated microglia (DAM), characterized by high expression of SPP1, CD63, TREM2, and APOE; (ii) lipid droplet-accumulating microglia (LDAM), marked by elevated ACSL1, NAMPT, DPYD, and CD163; and (iii) homeostatic microglia, defined by P2RY12, P2RY13, and CX3CR1 (**Figures 5H-J**). In APOE4/4 AD, LDAM were markedly expanded, whereas homeostatic microglia were reduced (**Figure 5K**). LDAM has been validated to promote AD-related pathology (20, 23). Our result showed that LDAM exhibited significantly increased IFN-γ pathway activity (**Figures 5L, M**), suggesting that IFN-γ may contribute to APOE4/4-driven AD pathology by promoting the expansion or maintenance of the LDAM population.

### 3.6 IFN-γ enhances ACSL1 expression in microglia

**Figure 6:**
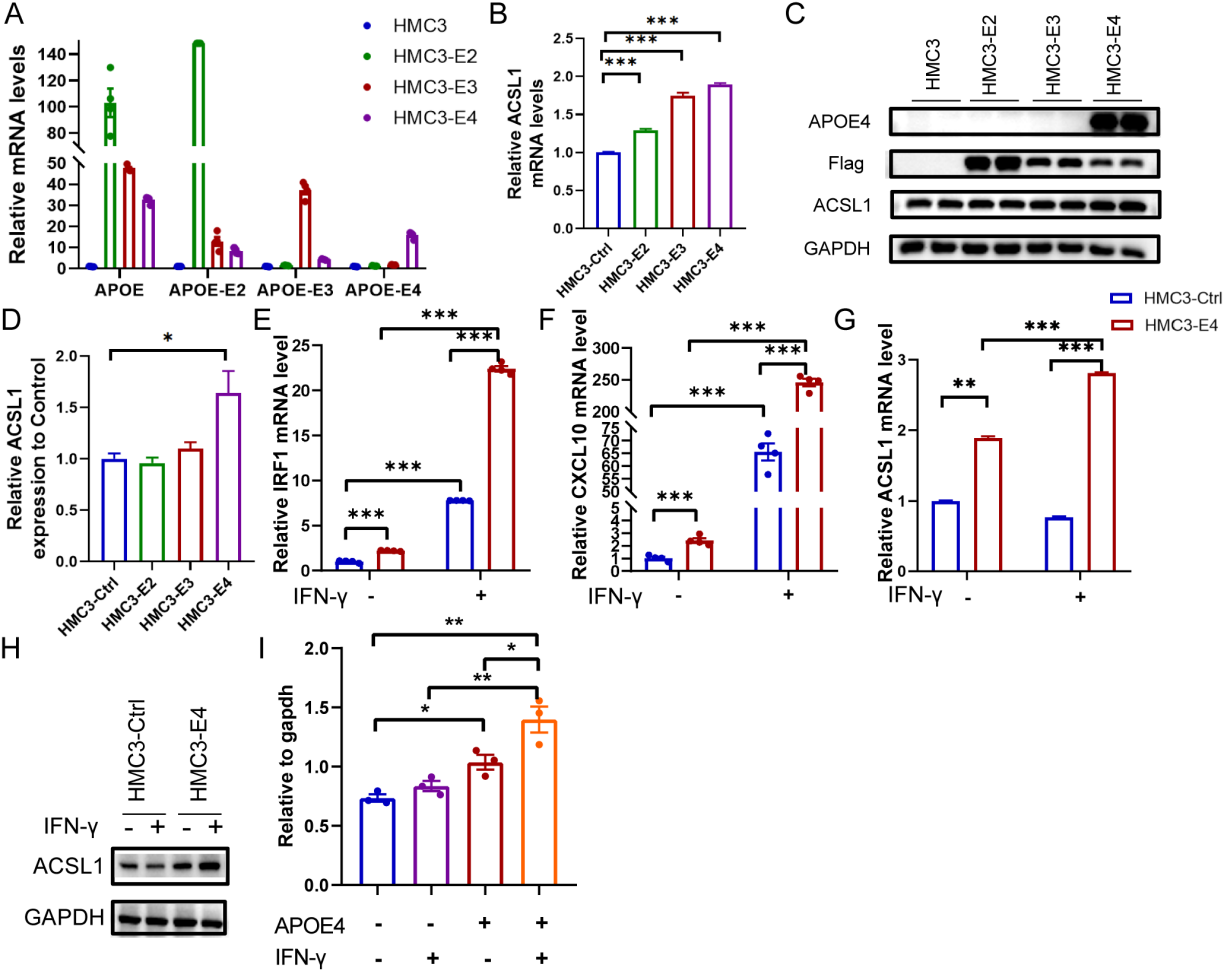
IFN-γ enhances ApoE4-induced ACSL1 expression in HMC3 microglial cells. **(A)** Quantitative PCR (qPCR) analysis of mRNA expression levels of APOE, APOE2, APOE3, and APOE4 in control HMC3 cells and HMC3 cells overexpressing ApoE2 (HMC3-E2), ApoE3 (HMC3-E3), or ApoE4 (HMC3-E4). **(B)** qPCR analysis of ACSL1 mRNA expression levels in control HMC3 cells, HMC3-E2, HMC3-E3, and HMC3-E4 cells. **(C)** Western blot analysis of ACSL1 protein expression in the indicated groups. **(D)** Quantification of ACSL1 protein levels shown in **(C)**. **(E, F)** qPCR analysis comparing the transcriptional expression levels of IRF1 and CXCL10 in control HMC3 cells and HMC3-E4 cells in the presence or absence of IFN-γ stimulation. **(G)** qPCR analysis of ACSL1 mRNA expression in control HMC3 cells and HMC3-E4 cells with or without IFN-γ stimulation. **(H)** Western blot analysis of ACSL1 protein expression under the indicated conditions. **(I)** Quantification of ACSL1 protein levels shown in **(H)**. *P < 0.05; **P < 0.01; ***P < 0.001.

To validate our findings, we first established HMC3 microglial cell lines stably overexpressing empty vector, ApoE2, ApoE3, or ApoE4 (**Figure 6A**). LDAMs are primarily defined by the expression of the lipid droplet-associated enzyme ACSL1, and overexpression of ACSL1 is sufficient to induce lipid droplet formation in multiple cell types, including microglia (20, 24). We confirmed that ApoE4 overexpression markedly increased both ACSL1 mRNA and protein levels in HMC3 cells (**Figures 6B-D**). We next evaluated whether IFN-γ modulates the LDAM phenotype by treating the cells with exogenous IFN-γ. As anticipated, IFN-γ stimulation robustly induced canonical IFN-γ-responsive genes, including CXCL10 and IRF1 (**Figures 6E, F**), indicating effective pathway activation in HMC3. Importantly, we found that IFN-γ further augmented ACSL1 transcription and protein expression specifically in ApoE4-overexpressing cells (**Figures 6G-I**). Together, these data demonstrate that IFN-γ amplified ACSL1 expression and promoted an ACSL1-driven LDAM phenotype in the context of ApoE4.

## 4 Discussion

Numerous studies have investigated plasma biomarkers associated with AD, leading to the proposal of multiple candidate protein markers. Among these, the Aβ42/Aβ40 ratio and p-tau residuals such as p-tau217 and p-tau231, although trivial in plasma, have emerged as particularly promising indicators of AD pathology and diagnosis with the development of technologies such as single molecule arrays (7, 25). Despite these advances, several challenges persist. First, optimal and standardized assays for their quantification have not yet been established. Head-to-head comparisons suggest that certain mass spectrometry-based methods outperform several immunoassays in detecting plasma Aβ42/Aβ40 ratio for inferring brain amyloid pathology, thus indicating the importance of choosing the appropriate methodology (26). In addition, clinically actionable cutoff thresholds have not been definitively defined (9, 25). Therefore, the identification of plasma biomarkers that enable early diagnosis, disease course prediction, or therapeutic monitoring for AD remains a major unmet clinical need.

In this study, we set out to identify plasma proteins, measurable using multiplex liquid-chip assays, that differentiated AD, MCI, and HC groups. We took as a starting point the powerful multisource evidence that inflammation dysregulation and brain-blood barrier compromise were critical components of AD pathogenesis (2, 27). In this context, profiling circulating inflammatory proteins may provide complementary diagnostic insights, capturing aspects of immune dysregulation not reflected by amyloid or p-tau. We measured 16 circulating proteins relevant to inflammation and observed pronounced alterations in AD patients. Specifically, IFN-γ, IL-33, and IL-18 were significantly elevated in AD patients compared with both MCI and HC groups, whereas IL-7 and CCL11 were markedly decreased in AD. Notably, we leveraged clinical factors, APOE genotype, and inflammatory markers to construct an integrated model with an AUC of 0.979, demonstrating superior predictive capacity for AD. Taken together, these findings revealed a complex pattern of marker changes in peripheral inflammation, which could be leveraged for AD diagnosis and therapeutic intervention.

Our findings highlight a dual aspect of immune dysregulation in AD. IL-18, a pro-inflammatory cytokine, was significantly increased in the plasma of AD patients. Similar elevations have been reported not only in peripheral blood but also in the brains of individuals with AD (28, 29). Mechanistically, IL-18 promotes amyloid-β production by upregulating glycogen synthase kinase-3β and induces tau hyperphosphorylation through activation of cyclin-dependent kinase 5, thereby contributing to key pathological features of AD (30–32). In contrast, IL-7 functions as a homeostatic cytokine that primarily supports the survival, development, and maintenance of lymphocytes (33). While a previous study reported no association between circulating IL-7 and dementia (34), we observed significantly decreased plasma IL-7 in AD patients compared with MCI or HC, consistent with another report showing lower plasma IL-7 in AD versus MCI (35). These results indicate compromised immune homeostasis that may facilitate AD conversion. Together, our data support a model in which AD pathogenesis involves simultaneous activation of pro-inflammatory pathways (e.g., IL-18) and disruption of immune homeostatic mechanisms (e.g., IL-7).

IL-33 is a key mediator of innate immunity and a regulator of immune cell infiltration and activation (36). Previous studies have reported conflicting changes in plasma IL-33 levels between AD patients and healthy controls (37, 38). Experimental evidence regarding the causal role of IL-33 in AD is also inconsistent. In AD transgenic mouse models, IL-33 administration enhanced microglial Aβ clearance, reduced proinflammatory responses, and promoted neuronal repair, yielding beneficial outcomes (39–41). Conversely, another study reported that IL-33 treatment impaired spatial memory and increased hippocampal inflammatory markers (42). Our findings provide additional evidence that elevated plasma IL-33 is associated with increased AD risk. However, it remains unclear whether this peripheral increase reflects CNS IL-33 levels or represents a compensatory immune response triggered by insufficiently effective IL-33 signaling during disease progression. Further studies are required to elucidate the molecular mechanisms underlying IL-33’s role in AD.

Our study demonstrated that plasma IFN-γ was markedly elevated in AD patients and negatively correlated with MMSE and MoCA scores, implicating IFN-γ as a risk factor for cognitive decline. These clinical observations are consistent with experimental evidence showing that IFN-γ induces microglial proliferation, synaptic loss, and nitric oxide release, leading to impaired synaptic transmission and disrupted γ-oscillatory activity, and ultimately cognitive dysfunction (43). *In vivo* evidence regarding the role of IFN-γ in AD remains dichotomous. In APP/PS1 mice, IFN-γ derived from Aβ-specific Th1 cells has been identified as a driver of microglial activation and plaque accumulation (44). Conversely, other studies using the APP/PS1 model suggest a protective function, demonstrating that IFN-γ enhances Aβ clearance via microglial autophagy (45). Distinct outcomes are observed in the 3xTg model where chronic IFN-γ exposure exacerbated amyloid pathology and inflammation, yet paradoxically reduced p-tau loads and increased neurogenesis (46). These discrepancies underscore that the impact of IFN-γ likely depends on the model system, the presence or absence of tau pathology, and the dose and duration of IFN-γ exposure.

Importantly, we further observed that IFN-γ was selectively elevated in APOE ϵ4 carriers diagnosed with AD, but not in those with MCI. This dissociation implies that the synergy between IFN-γ and APOE ϵ4 may not drive early disease pathology; rather, it appears to be a downstream event during the later progression of AD. Single-cell transcriptomic analyses of human brain tissue identified enrichment of IFN-γ signaling in microglia, particularly within the LDAM subcluster. Consistent with this observation, LDAM abundance was increased in APOE4/4 AD brains and has recently been shown to actively promote AD pathology (20, 23). Given that LDAM is characterized and driven by the key metabolic enzyme ACSL1, we demonstrated that IFN-γ treatment upregulated ACSL1 expression in ApoE4-overexpressing HMC3 microglia, thereby promoting the transition toward the LDAM phenotype. Collectively, our findings provide the first evidence linking IFN-γ signaling with APOE ϵ4-associated AD risk through modulation of the pathogenic LDAM microglial state. These findings suggest that plasma IFN-γ could serve as a potential biomarker for identifying APOE ϵ4 carriers at higher risk of AD, and that targeting the IFN-γ/ACSL1 axis may offer a novel therapeutic strategy to modulate pathogenic microglial states and slow disease progression.

Several limitations of this study should be acknowledged. First, the cross-sectional design of our clinical cohort precludes causal inference, and longitudinal studies, ideally with serial plasma and cerebrospinal fluid sampling, will be necessary to determine whether these changes predict disease onset or cognitive decline. Second, independent cohort validation is required to confirm the clinical findings. Third, although single-cell transcriptomic analyses and *in vitro* experiments support a mechanistic link between IFN-γ signaling and the LDAM microglial phenotype, direct *in vivo* evidence regarding the transport of peripheral IFN-γ across the blood-brain barrier to act on central microglia is warranted. Fourth, certain comorbidities and medication usages influence circulating inflammatory protein levels, potentially impacting the precision and interpretation of these measurements. Finally, the limited panel of plasma inflammatory proteins measured may not fully reflect the complexity of central nervous system inflammatory states during AD development.

## 5 Conclusion

In summary, our study demonstrates that multiple plasma inflammatory markers were significantly altered in AD when compared with MCI or HC individuals. Integrating clinical, single-cell transcriptomic, and *in vitro* data, we reveal that IFN-γ promotes the transition of microglia to the pathogenic LDAM phenotype via upregulation of ACSL1, proposing a mechanistic link between inflammation, APOE genotype, and microglial phenotype in AD.

## Data Availability

The raw data supporting the conclusions of this article will be made available by the authors, without undue reservation.
