# Supplementary material for: Identification of plasma inflammatory biomarkers for Alzheimer’s disease reveals IFN-γ as a regulator of ACSL1-mediated microglia phenotype
Source: Front Immunol. 2026 Feb 17;17:1770509. doi: 10.3389/fimmu.2026.1770509 (PMC12953125; doi:10.3389/fimmu.2026.1770509)
Supplement: Supplementary file 1 [file Table1.docx]

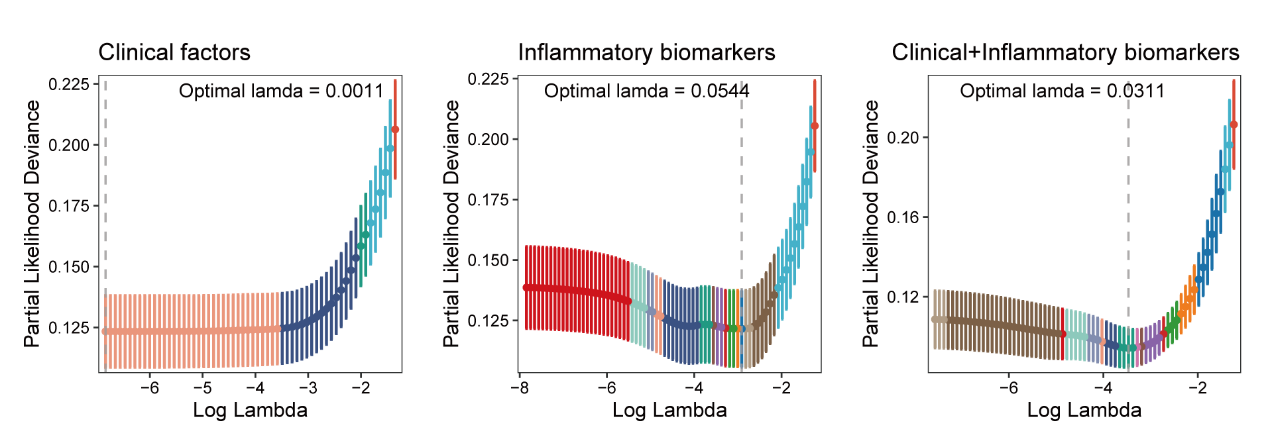


Figure S1. The optimal value of the regularization parameter (Lambda) was determined at the minimum partial likelihood deviance for the final models incorporating clinical factors, inflammatory markers, and their combination.


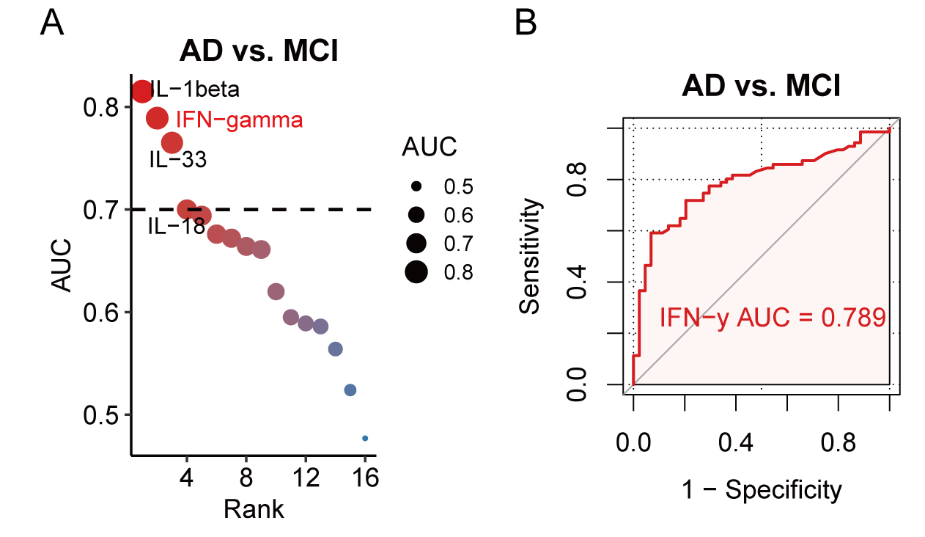


Figure S2. Predictive performance of 16 inflammatory proteins for distinguishing AD from MCI. (A) Results of Receiver operating characteristic (ROC) curve analysis evaluating the predictive performance of the 16 inflammatory proteins in distinguishing AD from MCI. (B) ROC curve analysis of IFN-γ for discriminating AD from MCI. AD, Alzheimer’s disease; MCI, mild cognitive impairment.
